# Comparison of CT-Determined Pulmonary Artery Diameter, Aortic Diameter, and Their Ratio in Healthy and Diverse Clinical Conditions

**DOI:** 10.1371/journal.pone.0126646

**Published:** 2015-05-08

**Authors:** Sang Hoon Lee, Young Jae Kim, Hye Jeong Lee, Hee Yeong Kim, Young Ae Kang, Moo Suk Park, Young Sam Kim, Se Kyu Kim, Joon Chang, Ji Ye Jung

**Affiliations:** 1 Division of Pulmonology, Department of Internal Medicine, Institute of Chest Diseases, Severance Hospital, Yonsei University College of Medicine, Seoul, Republic of Korea; 2 Department of Radiology, Severance Hospital, Yonsei University College of Medicine, Seoul, Republic of Korea; 3 Department of Radiology, Kangnam Sacred Heart Hospital, Hallym University College of Medicine, Seoul, Republic of Korea; Nagoya University, JAPAN

## Abstract

**Background:**

The main pulmonary artery diameter (mPA), aortic diameter (Ao), and the mPA/Ao ratio, easily measured using chest computed tomography (CT), provide information that enables the diagnosis and evaluation of cardiopulmonary diseases. Here, we used CT to determine the sex- and age-specific distribution of normal reference values for mPA, Ao, and mPA/Ao ratio in an adult Korean population.

**Methods:**

Data from non-contrast, ECG-gated, coronary-calcium-scoring CT images of 2,547 individuals who visited the Health Screening Center of the Severance Hospital were analyzed. Healthy individuals (n = 813) included those who do not have hypertension, diabetes, asthma, obstructive lung disease, ischemic heart disease, stroke, smoking, obesity, and abnormal CT findings. Both mPA and Ao were measured at the level of bifurcation of the main pulmonary artery.

**Results:**

The mean mPA and Ao were 25.9 mm and 30.0 mm in healthy participants, respectively, while the mean mPA/Ao ratio was 0.87. Medical conditions associated with a larger mPA were male, obesity, smoking history, hypertension, and diabetes. A larger mPA/Ao ratio was associated with female, the obese, non-smoker, normotensive, and normal serum level of lipids, while a smaller mPA/Ao ratio was associated with older age. In healthy individuals, the 90th percentile sex-specific mPA, Ao, and mPA/Ao ratio were, 31.3 mm (95% CI 29.9–32.2), 36.8 mm (95% CI 35.7–37.5), and 1.05 (95% CI 0.99–1.07) in males, and 29.6 mm (95% CI 29.1–30.2), 34.5 mm (95% CI 34.1–34.9), and 1.03 (95% CI 1.02–1.06) in females, respectively.

**Conclusion:**

In the Korean population, the mean mPA reference values in male and female were 26.5 mm and 25.8 mm, respectively, while the mean mPA/Ao ratio was 0.87. These values were influenced by a variety of underlying medical conditions.

## Introduction

Chest computed tomography (CT) is widely performed in patients with respiratory symptoms because of its simplicity and accessibility, although radiation exposure is a concern. Among the various types of clinical findings provided by chest CT, the main pulmonary artery diameter (mPA) and aortic diameter (Ao) are readily measurable.

The mPA, as determined by chest CT, is a reliable predictor of pulmonary hypertension [1,2], which is defined as a resting mean pulmonary artery pressure ≥ 25 mmHg, measured by right cardiac catheterization. Enlargement of the pulmonary artery is associated with the severe exacerbations of chronic obstructive pulmonary disease (COPD) [3,4]. Recently, Nakanishi et al. [5] reported that an elevated (> 0.9) pulmonary trunk diameter to ascending aorta diameter ratio was significantly associated with an increased risk of all-cause death, regardless of the presence or absence of coronary artery disease. Therefore, mPA and the ratio of mPA to aortic diameter (mPA/Ao ratio) can inform clinicians on the risk and current status of a diverse range of underlying illnesses, as well as provide patient prognostic information.

However, to determine the clinical significance of mPA and mPA/Ao ratio in cardiopulmonary disease, normal reference values for mPA, Ao, and mPA/Ao ratio must be defined, and the different clinical characteristics associated with an elevated versus normal mPA/Ao ratio should be determined.

Although mPA has been previously measured using chest radiography or chest CT [5–7], the sample sizes of those studies were insufficient to determine normal reference values, especially in Asian countries such as Japan and Turkey [5,7]. Moreover, in Korea, large population studies to establish the normal reference ranges of mPA and investigate the relationship between mPA, mPA/Ao ratio, and clinical parameters are lacking [6–8]. Thus, in this study, we aimed to examine the sex- and age- specific distribution of mPA, Ao, and mPA/Ao ratio using ECG-gated coronary-calcium-scoring CT to establish normal reference values in a Korean population. We also investigated the relationship between these values and various clinical conditions.

## Methods

### Study participants

In this retrospective single-center study, we examined 2,547 individuals (1,023 females, 1,524 males) who visited the Severance Hospital Health Screening Center, Seoul, Korea, between January 2011 and December 2012. Demographic, underlying diseases, lung function, transthoracic echocardiography (TTE), and CT imaging data were obtained from electronic medical records. Individuals who were < 20 years of age, pregnant, or had a history of congenital heart disease, valvular heart disease, thoracic surgery, or radiotherapy, were excluded. Normal reference values were obtained from a group of healthy individuals (n = 813), defined as those who do not have hypertension, diabetes mellitus (DM), asthma, obstructive lung disease (OLD), ischemic heart disease (IHD), stroke, smoking history, obesity, or abnormal pulmonary CT findings. This study was reviewed and approved by the Yonsei University Health Service, Severance Hospital Institutional Review Board (Ref. 4-2013-0839). A written informed consent was not necessary because of the retrospective cohort nature of the study.

### Definitions

Obesity and OLD were defined as a body mass index (BMI) > 30 kg/m^2^, and as a ratio of forced expiratory volume in 1 second to forced vital capacity (FEV_1_/FVC) < 70%, according to a pulmonary function test, respectively. Abnormal pulmonary CT findings included bronchiectasis, interstitial lung disease, emphysema, bronchial thickening, any mass, or fibrosis.

### CT image acquisition

Non-contrast, ECG-gated, coronary-calcium-scoring CT was performed using a 64-slice multi-detector CT (LightSpeed Volume CT, GE Healthcare, Waukesha, Wisconsin). Prospective ECG-triggered acquisitions were obtained in mid-diastole at 120–140 kVp and 150–220 mA, depending on the patient’s size. Other parameters were a 350 ms gantry rotation time and a 64 × 1.25 mm slice collimation. The CT was reconstructed at 70% of the R-R interval using a slice thickness of 2.5 mm and an increment of 2.5 mm.

### Measurement of the pulmonary artery and aortic diameters

**Fig 1:**
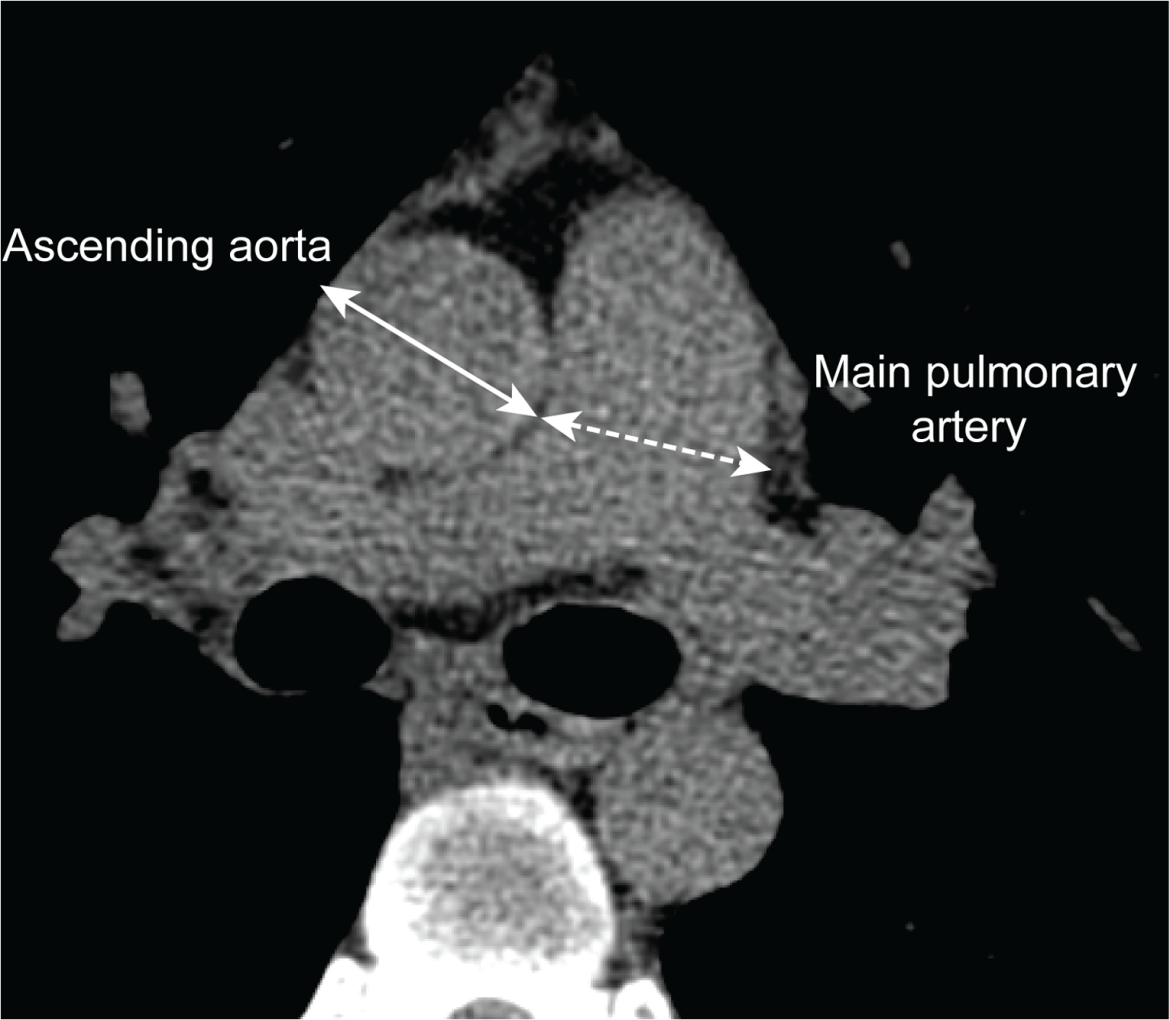
Measurement of the pulmonary artery and ascending aorta diameters. Pulmonary artery and ascending aorta diameters were assessed on a transverse CT image at the level of the pulmonary artery bifurcation. Vessel diameters were obtained by measuring the widest diameter vertical to the long axis of the main pulmonary artery, using a computer caliper. mPA, main pulmonary artery; Ao, aorta

The pulmonary artery and ascending aorta were assessed on a transverse image at the level of the pulmonary artery bifurcation. Vessel diameters were obtained by measuring the widest diameter vertical to the long axis of the main pulmonary artery, using a computer caliper (Fig 1). A chest radiologist blinded to the study design and clinical data reviewed all the CT images, and measured the mPA and Ao at a workstation. Interobserver agreement was determined by comparing the mPA and Ao measurements with those obtained from a second blinded pulmonologist at a different workstation. The κ value of the intraclass correlation coefficient was 0.95 (95% confidence interval (CI), 0.94–0.95) for mPA, and 0.97 (95% CI, 0.96–0.97) for Ao.

### Transthoracic echocardiography

TTE data were available from 289 individuals, of whom, 69 were healthy. TTE was performed using a Vivid 7 apparatus (GE Medical Systems, Milwaukee, WI, USA) and data collected included left ventricular end diastolic diameter (LVEDD), left ventricular end systolic diameter (LVESD), left ventricular ejection fraction (LVEF), left atrial volume index (LAVI), and right ventricular systolic pressure (RVSP).

### Statistical analysis

Continuous variables were analyzed using Student’s *t*-test or Mann-Whitney U test. Categorical variables were analyzed using Pearson χ-square test or Fisher’s exact test. mPA, Ao, and mPA/Ao ratio values at the 10^th^, 25^th^, 50^th^, 75^th^, and 90^th^ percentiles were derived from the healthy reference group (n = 813). Pearson’s correlation analyses were performed to examine relationships between age, BSA, FEV_1_, echocardiography data; and mPA, Ao, and mPA/Ao ratio in all participants. Multiple linear regression analysis was conducted to determine the effect of demographic factors, underlying diseases, and lung function on mPA, Ao, and mPA/Ao ratio. All statistical analyses including 95% CI for the 90^th^ percentile values were conducted using the SPSS version 20.0 software (SPSS, Chicago, IL, USA). A two-sided *P*-value < 0.05 was considered statistically significant for all analyses.

## Results

### Baseline characteristics

**Fig 2:**
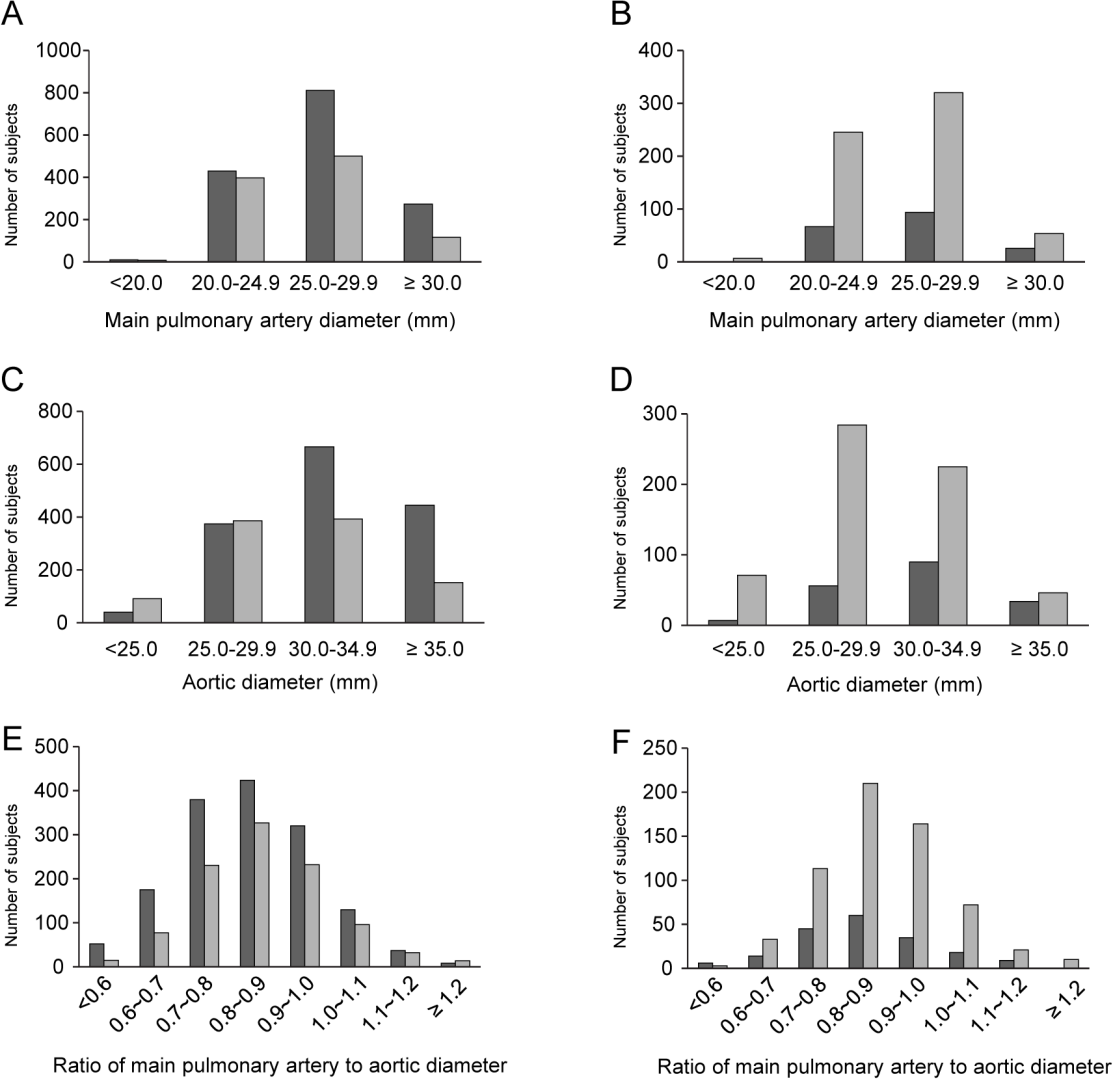
Distribution of mPA, Ao, and mPA/Ao ratio. Histograms show the distributions of mPA, Ao and mPA/Ao ratio values in all (n = 2,547) (A, C, E) and healthy participants (n = 813) (B, D, F), respectively (dark gray: male, gray: female). mPA, main pulmonary artery; Ao, aorta.

Table 1 shows the baseline clinical characteristics of all participants (n = 2,547) and healthy individuals (n = 813, 187 males and 626 females), and their distribution according to age and BMI (S1 Fig) (Details are shown in S1 Table). The mean age of the entire group was 53.1 years, and that of the healthy group was 51.2 years. The body surface area (BSA) was 1.74 and 1.63, respectively. Of all participants, 3.9% were obese and 50.2% had a history of smoking. Underlying diseases included hypertension (23.7%), DM (8.5%), dyslipidemia (4.9%), asthma (0.4%), IHD (0.6%), stroke (0.5%), and OLD (2.6%). The overall mean mPA, Ao, and mPA/Ao ratio in all participants were 26.6 mm, 32.0 mm, and 0.84, respectively, while in the healthy reference group, the corresponding values were 25.9 mm, 30.0 mm, and 0.87, respectively. Fig 2 shows the mPA, Ao, and mPA/Ao ratio distributions in all participants and healthy participants, with mean values of RVSP of 24.1 mmHg, and 24.9 mmHg, respectively.

**Table 1:** Baseline characteristics of the study population. ^*^

	Total participants	Healthy reference group
	(n = 2,547)	(n = 813)
Age, yr	53.1 ± 9.3	51.2 ± 8.8
M:F (%)	1,524:1,023 (59.8:40.2)	187:626 (23.0:77.0)
BMI, kg/m^2^	24.2 ± 3.1	23.1 ± 2.6
BSA, m^2^	1.74 ± 0.18	1.63 ± 0.15
Height, cm	165.1 ± 8.4	160.7 ± 7.3
Weight, kg	66.2 ± 11.6	59.9 ± 8.9
Obesity^†^	100 (3.9)	-
SBP, mmHg	119.6 ± 14.4	115.8 ± 14.5
DBP, mmHg	78.0 ± 10.7	74.3 ± 10.7
Smoking history		
Current	546 (22.1)	-
Former	696 (28.1)	-
Never	1,232 (49.8)	-
Hypertension	603 (23.7)	-
DM	217 (8.5)	-
Dyslipidemia	125 (4.9)	-
Asthma	9 (0.4)	-
IHD	16 (0.6)	-
Stroke	14 (0.5)	-
OLD^‡^	65 (2.6)	-
mPA diameter, mm	26.6 ± 3.4	25.9 ± 3.2
Aortic diameter, mm	32.0 ± 4.5	30.0 ± 4.0
mPA/Ao ratio	0.84 ± 0.13	0.87 ± 0.13
FVC, L	3.44 ± 0.77	3.10 ± 0.68
FVC,% predicted	91.2 ± 10.5	92.9 ± 10.1
FEV_1_, L	2.86 ± 0.66	2.6 ± 0.58
FEV_1_,% predicted	102.2 ± 13.4	104.6 ± 12.3
FEV_1_/FVC,%	83.1 ± 6.3	84.6 ± 5.5
Echocardiography^§^		
LVEDD, mm	48.6 ± 3.7	47.5 ± 3.7
LVESD, mm	31.4 ± 3.6	30.8 ±3.4
LVEF, %	67.7 ± 5.9	67.6 ±5.3
LAVI	23.7 ± 7.8	23.5 ±9.0
RVSP, mmHg	24.1 ± 5.5	24.9 ±6.8

^*^ The data are the numerical values, with the percentages in parentheses; or the mean ± standard deviation, unless otherwise indicated.

^†^ Obesity was defined as a BMI > 30 kg/m^2^.

^‡^ OLD was defined as FEV_1_/FVC (%) < 70%.

^§^ Data were available in 289 of total subjects and 69 in healthy subjects.

BMI, body mass index; BSA, body surface area; SBP, systolic blood pressure; DBP, diastolic blood pressure; DM, diabetes mellitus; IHD, ischemic heart disease; OLD, obstructive lung disease; mPA, main pulmonary artery; Ao, aorta; FVC, forced vital capacity; FEV_1_, forced expiratory volume in 1 second; LVEDD, left ventricular end diastolic diameter; LVESD, left ventricular end systolic diameter; LVEF, left ventricular ejection fraction; LAVI, left atrial volume index; RVSP, right ventricular systolic pressure

### mPA, Ao, and mPA/Ao ratio in diverse clinical conditions

Table 2 shows the relationship between mPA, Ao, and mPA/Ao ratio according to sex, obesity, smoking status, hypertension, DM, dyslipidemia, asthma, IHD, stroke, OLD, and abnormal pulmonary findings on chest CT. The mPA was significantly larger in males (27.0 mm vs. 26.0 mm, *P* < 0.001), the obese (29.3 mm vs. 26.5 mm, *P* < 0.001), and in those with a history of smoking (26.9 mm vs. 26.4 mm, *P* < 0.001), hypertension (27.6 mm vs. 26.3 mm, *P* < 0.001), or DM (28.2 mm vs. 26.5 mm, *P* < 0.001). While a similar trend was observed for Ao, this diameter was also significantly larger in individuals with dyslipidemia, stroke, OLD, or abnormal pulmonary findings on chest CT. The mPA/Ao ratio was significantly larger in females (0.86 vs. 0.83, *P* < 0.001), the obese (0.89 vs. 0.84, *P* < 0.001), or in those with abnormal findings on chest CT (0.85 vs. 0.81, *P* = 0.002), but significantly smaller in those with hypertension (0.80 vs. 0.86, *P* < 0.001), a history of smoking (0.84 vs. 0.85, *P* = 0.002), or dyslipidemia (0.79 vs. 0.85, *P* < 0.001).

**Table 2:** Comparison of mPA, Ao, and mPA/Ao in the study population according to clinical condition (n = 2,547). ^*^

	mPA (mm)	*P*-value	Ao (mm)	*P*-value	mPA/Ao ratio	*P*-value
Sex	Female (1,023)	26.0 ± 3.4	< 0.001	30.7 ± 4.4	< 0.001	0.86 ± 0.13	< 0.001
	Male (1,524)	27.0 ± 3.4		32.8 ± 4.4		0.83 ± 0.13	
Obesity^†^	Absence (2,447)	26.5 ± 3.3	< 0.001	31.9 ± 4.5	0.005	0.84 ± 0.13	< 0.001
	Presence (100)	29.3 ± 4.0		33.2 ± 4.7		0.89 ± 0.13	
Smoking	Never (1,232)	26.4 ± 3.4	< 0.001	31.3 ± 4.5	< 0.001	0.85 ± 0.13	0.002
	Ever (1,242)	26.9 ± 3.4		32.6 ± 4.5		0.84 ± 0.13	
Hypertension	Absence (1,944)	26.3 ± 3.3	< 0.001	31.1 ± 4.3	< 0.001	0.86 ± 0.13	< 0.001
	Presence (603)	27.6 ± 3.6		34.7 ± 4.2		0.80 ± 0.12	
DM	Absence (2,330)	26.5 ± 3.4	< 0.001	31.8 ± 4.5	< 0.001	0.85 ± 0.13	0.433
	Presence (217)	28.2 ± 3.5		34.0 ± 4.0		0.84 ± 0.13	
Dyslipidemia	Absence (2,422)	26.6 ± 3.4	0.805	31.8 ± 4.5	< 0.001	0.85 ± 0.13	< 0.001
	Presence (125)	26.7 ± 3.8		34.1 ± 4.2		0.79 ± 0.13	
Asthma	Absence (2,538)	26.6 ± 3.4	0.227	32.0 ± 4.5	0.823	0.85 ± 0.13	0.453
	Presence (9)	25.3 ± 2.0		31.4 ± 3.7		0.82 ± 0.14	
IHD	Absence (2,531)	26.6 ± 3.4	0.138	31.9 ± 4.5	0.078	0.84 ± 0.13	0.613
	Presence (16)	28.0 ± 4.1		33.9 ± 3.8		0.83 ± 0.12	
Stroke	Absence (2,533)	26.6 ± 3.4	0.422	31.9 ± 4.5	0.036	0.85 ± 0.13	0.116
	Presence (14)	27.1 ± 2.4		34.4 ± 4.1		0.79 ± 0.09	
OLD^‡^	Absence (2,440)	26.6 ± 3.4	0.067	31.9 ± 4.5	0.001	0.85 ± 0.13	0.198
	Presence (65)	27.6 ± 4.2		33.8 ± 4.2		0.82 ± 0.14	
Abnormal CT	Absence (2,381)	26.6 ± 3.4	0.070	31.8 ± 4.5	< 0.001	0.85 ± 0.13	0.002
finding	Presence (166)	27.1 ± 3.4		33.9 ± 5.0		0.81 ± 0.14	
Bronchiectasis	Absence (2,483)	26.6 ± 3.4	0.302	31.9 ± 4.5	< 0.001	0.85 ± 0.13	0.063
	Presence (64)	27.0 ± 3.3		33.8 ± 5.0		0.81 ± 0.15	
Emphysema	Absence (2,489)	26.6 ± 3.4	0.252	31.9 ± 4.5	0.002	0.85 ± 0.13	0.098
	Presence (58)	27.1 ± 3.6		33.7 ± 4.7		0.82 ± 0.14	
Fibrosis	Absence (2,505)	26.6 ± 3.4	0.548	31.9 ± 4.5	0.003	0.85 ± 0.13	0.036
	Presence (42)	26.9 ± 3.1		34.0 ± 5.0		0.80 ± 0.11	

^*^ The number in parentheses is the number of affected individuals. Other values are the mean ± standard deviation.

^†^ Obesity was defined as BMI > 30 kg/m^2^.

^‡^ OLD was defined as FEV_1_/FVC (%) < 70%.

mPA, main pulmonary artery; Ao, aorta; DM, diabetes mellitus; IHD, ischemic heart disease; OLD, obstructive lung disease; CT, computed tomography

Correlations between mPA, Ao, and mPA/Ao ratio with age, BSA, and FEV_1_ (%) are shown in S2 Fig. Age was significantly correlated with mPA, Ao, and mPA/Ao ratio. BSA was correlated with mPA and Ao, and FEV_1_ (%) with mPA and mPA/Ao ratio. Correlations between mPA, Ao, and mPA/Ao ratio with LAVI and RVSP are shown in S3 Fig. LAVI was significantly correlated with mPA and Ao, but RVSP showed no correlation with any of the three parameters.

### mPA, Ao, and mPA/Ao ratio in the healthy reference group

The distributions of mean mPA, Ao, and mPA/Ao ratio for the healthy participants are shown in Table 3 according to age. Mean mPA values in males and females ranged from 26.0–27.0 mm, and 24.4–27.0 mm, respectively. A larger age-related difference in mPA was observed in females than in males (2.6 mm vs. 1.0 mm). Although the mPA was larger in males than in females for all percentiles and for ages < 45 and 45–54 years, the opposite was true for females age ≥ 55 years. The 90^th^ percentile value of mPA was 31.3 mm (95% CI 29.9–32.2) in males and 29.6 mm (95% CI 29.1–30.2) in females. Mean Ao was 29.4–33.8 mm in males and 26.4–31.8 mm in females. The difference in mean Ao was greater in females than in males (5.4 mm vs. 4.4 mm). Aortic enlargement was observed with aging. The mean mPA/Ao ratio was larger in females than in males for all age groups, but tended to decrease with increasing age in both sexes. The 90^th^ percentile value of mPA/Ao ratio was 1.05 (95% CI 0.99–1.07) in males and 1.03 (95% CI 1.02–1.06) in females.

**Table 3:** mPA, Ao and mPA/Ao according to age in healthy reference group (n = 813).

Variable		Men (n = 187)				Women (n = 626)			
mPA,	Age, yr	<45	45–54	≥55	total	<45	45–54	≥55	total
mm	n	52	73	62	187	124	286	216	626
	Mean	26.0	27.0	26.3	26.5	24.4	25.4	27.0	25.8
	SEM	0.40	0.43	0.45	0.25	0.24	0.16	0.22	0.12
	10th	22.3	22.2	21.8	22.2	21.3	21.5	23.3	21.7
	25th	24.2	24.1	23.9	24.1	22.3	23.5	24.9	23.8
	50th	25.5	26.9	26.0	26.1	24.4	25.5	26.5	25.8
	75th	27.7	29.8	27.5	28.6	26.3	27.2	28.7	27.5
	90th	29.1	31.8	31.4	31.3	27.6	29.0	31.5	29.6
Ao,	Mean	29.4	31.3	33.8	31.6	26.4	29.3	31.8	29.6
mm	SEM	0.49	0.39	0.50	0.29	0.27	0.20	0.23	0.15
	10th	25.4	26.9	29.3	26.5	22.8	25.0	28.1	24.5
	25th	26.5	29.2	30.7	29.0	24.1	27.0	29.6	27.0
	50th	29.1	31.7	32.7	31.6	26.5	29.0	31.4	29.5
	75th	32.1	33.6	36.1	34.1	28.0	31.6	33.8	31.9
	90th	34.4	36.2	40.3	36.8	29.9	33.9	36.4	34.5
mPA/Ao	Mean	0.90	0.87	0.79	0.85	0.93	0.88	0.86	0.88
ratio	SEM	0.017	0.015	0.018	0.01	0.011	0.007	0.008	0.005
	10th	0.74	0.71	0.61	0.69	0.80	0.73	0.72	0.74
	25th	0.80	0.78	0.70	0.76	0.86	0.80	0.77	0.80
	50th	0.86	0.85	0.79	0.83	0.92	0.87	0.85	0.87
	75th	0.98	0.94	0.85	0.94	1.00	0.96	0.92	0.96
	90th	1.08	1.06	0.97	1.05	1.09	1.02	1.02	1.03

mPA, main pulmonary artery; Ao, aorta; SEM, standard error of the mean

### Clinical characteristics of the groups with mPA/Ao ratio < 1.04 and mPA/Ao ratio ≥ 1.04

Clinical characteristics were compared based on the 90^th^ percentile mPA/Ao ratio value of 1.04 as shown in Table 4. Individuals with mPA/Ao ratio **≥** 1.04 were significantly younger, predominantly female, and less likely to have hypertension. They also had a smaller FVC (%) and FEV_1_ (%), and a larger FEV_1_/FVC. LAVI was significantly larger in participants with mPA/Ao ratio **<** 1.04, while RVSP did not differ between the groups.

**Table 4:** Clinical characteristics of the groups with mPA/Ao ratio < 1.04 and mPA/Ao ratio ≥ 1.04 (n = 2,547). ^*^

	mPA/Ao ratio < 1.04	mPA/Ao ratio ≥ 1.04	*P*-value
	(n = 2,363)	(n = 184)	
Age, yr	53.6 ± 9.1	47.4 ± 9.6	<0.001
Male (%)	1,427 (60.4)	97 (52.7)	0.041
BMI, kg/m^2^	24.2 ± 3.0	23.9 ± 3.6	0.113
BSA, m^2^	1.74 ± 0.18	1.75 ± 0.21	0.637
Height, cm	165.0 ± 8.3	166.3 ± 8.6	0.035
Weight, kg	66.2 ± 11.4	66.4 ± 13.6	0.875
Obesity^†^	88 (3.7)	12 (6.5)	0.060
Smoking history			0.212
Current	507 (22.1)	39 (21.4)	
Former	654 (28.5)	42 (23.1)	
Never	1,131 (49.3)	101 (55.5)	
Hypertension	583 (24.7)	20 (10.9)	<0.001
DM	205 (8.7)	12 (6.5)	0.313
Dyslipidemia	121 (5.1)	4 (2.2)	0.075
Asthma	8 (0.3)	1 (0.5)	0.491
IHD	15 (0.6)	1 (0.5)	1.000
Stroke	14 (0.6)	0 (0.0)	0.617
OLD^†^	61 (2.6)	4 (2.2)	1.000
mPA diameter, mm	26.4 ± 3.2	30.0 ± 3.8	<0.001
Aorta diameter, mm	32.3 ± 4.4	27.0 ± 3.5	<0.001
mPA/Ao ratio	0.82 ± 0.11	1.11 ± 0.07	<0.001
FVC, L	3.44 ± 0.76	3.50 ± 0.80	0.268
FVC, % predicted	91.4 ± 10.5	88.9 ± 10.7	0.002
FEV_1_, L	2.85 ± 0.65	2.96 ± 0.69	0.042
FEV_1_, % predicted	102.5 ± 13.4	99.2 ± 12.5	0.002
FEV_1_/FVC, %	83.0 ± 6.4	84.5 ± 5.6	0.001
Abnormal CT findings	156 (6.6)	10 (6.0)	
Bronchiectasis	59 (2.5)	5 (2.7)	0.806
Emphysema	56 (2.4)	2 (1.1)	0.436
Fibrosis	42 (1.8)	0 (0.0)	0.070
Others	18 (0.8)	4 (2.2)	0.069
Echocardiography^§^			
LVEDD, mm	48.7 ± 3.7	47.8 ± 3.9	0.177
LVESD, mm	31.5 ± 3.7	30.5 ± 3.2	0.139
LVEF, %	67.5 ± 6.1	68.8 ± 4.9	0.229
LAVI	24.1 ± 7.7	20.9 ± 8.2	0.022
RVSP, mmHg	24.0 ± 5.6	25.1 ± 5.0	0.303

^*^ The data are the numerical values, with the percentages in parentheses; or the mean ± standard deviation, unless otherwise indicated.

^†^ Obesity was defined as body mass index > 30 kg/m^2^.

^‡^ OLD was defined as FEV_1_/FVC (%) < 70%.

^§^ Data were available in 253 of subjects with mPA/Ao ratio < 1.04 and 36 in subjects with mPA/Ao ratio ≥ 1.04.

BMI, body mass index; BSA, body surface area; DM, diabetes mellitus; IHD, ischemic heart disease; OLD, obstructive lung disease; mPA, main pulmonary artery; Ao, aorta; FVC, forced vital capacity; FEV_1_, forced expiratory volume in 1 second; CT, computed tomography; LVEDD, left ventricular end diastolic diameter; LVESD, left ventricular end systolic diameter; LVEF, left ventricular ejection fraction; LAVI, left atrial volume index; RVSP, right ventricular systolic pressure

### Multivariate linear analysis of the effects of clinical characteristics on mPA, Ao, and mPA/Ao ratio

The association between mPA, Ao, and mPA/Ao ratio with various demographic factors, underlying diseases, and physiologic parameters was analyzed using a multiple linear regression model (Table 5). The mPA/Ao ratio was positively associated with BSA, obesity, and DM, but negatively associated with age, male (vs. female) sex, current smoking, hypertension, dyslipidemia, and FEV_1_ (%).

**Table 5:** Multivariate linear analysis for the effects of clinical characteristics on mPA, Ao, and mPA/Ao ratio.

Variables	mPA			Ao			mPA/Ao ratio		
	Estimated	SE	*P*-value	Estimated	SE	*P*-value	Estimated	SE	*P*-value
Age, yr	0.08	0.008	< 0.001	0.25	0.008	< 0.001	-0.004	0.000	<0.001
Male	-1.06	0.227	< 0.001	0.29	0.254	0.250	-0.040	0.009	<0.001
BSA, m^2^	7.92	0.543	<0.001	6.79	0.608	<0.001	0.063	0.021	0.003
Obesity^*^	0.74	0.361	0.040	-0.54	0.404	0.179	0.034	0.014	0.016
Smoking									
Non smoker	Reference			Reference			Reference		
Ex-smoker	-0.20	0.193	0.298	-0.12	0.216	0.587	-0.002	0.008	0.797
Current smoker	-0.39	0.205	0.078	0.19	0.229	0.401	-0.016	0.008	0.048
Hypertension	0.40	0.159	0.014	1.81	0.178	< 0.001	-0.032	0.006	<0.001
DM	0.95	0.231	< 0.001	0.47	0.259	0.854	0.026	0.009	0.004
Dyslipidemia	-0.33	0.301	0.278	0.92	0.338	0.007	-0.035	0.012	0.003
Asthma	-0.77	1.042	0.460	0.25	1.168	0.828	-0.031	0.041	0.446
IHD	0.63	0.811	0.438	-0.81	0.909	0.375	0.032	0.032	0.315
Stroke	-0.60	0.871	0.494	0.65	0.976	0.505	-0.036	0.034	0.290
FEV_1_, % predicted	-0.01	0.005	0.004	0.03	0.005	0.625	-0.001	0.000	0.004

^*^ Obesity was defined as body mass index > 30 kg/m^2^.

mPA, main pulmonary artery; Ao, aorta; SE, standard error; BSA, body surface area; DM, diabetes mellitus; IHD, ischemic heart disease; FEV_1_, forced expiratory volume in 1 second

## Discussion

We used non-contrast, ECG-gated coronary-calcium-scoring CT scans to obtain reference values for mPA and Ao in the Korean population, and then determined the relationship between these values and various clinical conditions. Both mPA and Ao were larger in males, the obese, and those with hypertension, DM, or dyslipidemia. Larger mPA/Ao ratio was found in females, the obese, non-smokers, normotensive and in individuals with normal serum level of lipids. In the healthy reference group, mean mPA, Ao, and mPA/Ao ratio were 26.5 mm, 31.6 mm, and 0.85 in males, and 25.8 mm, 29.6 mm, and 0.88 in females, respectively. The 90^th^ percentile sex-specific values for mPA, Ao, and mPA/Ao ratio were 31.3 mm, 36.8 mm, and 1.05 in males, and 29.6 mm, 34.5 mm, and 1.03 in females, respectively. Factors influencing mPA diameter were age, sex, BSA, obesity, hypertension, DM, and FEV_1_ (%), whereas those influencing Ao were age, BSA, hypertension, and dyslipidemia. The mPA/Ao ratio was affected by age, sex, BSA, obesity, current smoking, hypertension, DM, dyslipidemia, and FEV_1_ (%).

All three parameters (i.e., mPA, Ao, and mPA/Ao ratio) are easily obtained through chest or heart CT images, and were previously shown to clinically correlate with underlying cardiopulmonary diseases [3,4,6,9,10]. In addition, Matsushita et al. [11] reported a significant difference in mPA between patients with stable interstitial pneumonia and those with acute disease exacerbation. Similarly, we found significant differences in mPA, Ao, and mPA/Ao ratio according to sex, obesity, smoking status, hypertension, DM, dyslipidemia, stroke, OLD, and abnormal CT findings. Male sex and any of these clinical conditions are risk factors for cardiopulmonary disease, which explains their positive relationship with mPA and Ao. By contrast, in the healthy reference group, aging was associated with a lower mPA/Ao ratio due to aortic enlargement. In this study, age was positively associated with mPA and Ao, but the effect of age was larger on Ao than on mPA.

In previous studies in which chest CT was used to investigate mPA in a reference group, the mean mPA ranged from 24.2 mm to 27.2 mm [6–8], and the mean mPA of 25.9 mm was found in our group of healthy individuals. The mean Ao in our reference group was 31.6 mm in males and 29.6 mm in females (i.e., slightly lower than the values reported by Rogers et al. [12] [34.1 and 31.9, respectively]). This difference can be explained by differences in the study populations who showed relatively higher BMI or BSA, and more medical problems in the study of Rogers et al. [12] Mao et al. [13] reported a mean Ao of 33.6 mm in males and 31.1 mm in females. Their study population also had a relatively higher mean BSA than that in our study. These factors, in addition to ethnic and lifestyle differences, may account for the differences in Ao values.

The 90^th^ percentile sex-specific mPA, Ao, and mPA/Ao ratio values determined in our healthy reference group were, 31.3 mm, 36.8 mm, and 1.05 in males, and 29.6 mm, 34.5 mm, and 1.03 in females, respectively. In several studies, mPA cutoff values ranging from 28.6 mm to 33.2 mm have been reported for the prediction of pulmonary hypertension [6,8,10]. However, in our study, the 90^th^ percentile mPA/Ao ratio value of 1.04, was greater than the previously reported ratios of 0.9–1.0 [3,5,14,15]. This difference may reflect the exclusion from the healthy reference group of individuals with abnormal CT findings, which are associated with a significantly lower mPA/Ao ratio (Table 2). The group with mPA/Ao ratio < 1.04 was older, had aortic enlargement, and a higher prevalence of hypertension. These comorbidities are among the cardiovascular diseases primarily affecting Ao rather than mPA. In a linear regression analysis, hypertension, and dyslipidemia were positively correlated with Ao, and the effect of hypertension was greater on Ao than on mPA (Table 5).

Blum et al. [16] reported that, compared to a healthy control group, COPD patients had a more dilated brachial artery and a smaller change in post-hyperemia arterial diameter. Iyer et al. [17] reported that mPA/Ao ratio > 1.0, as determined on CT scan, was better than echocardiography for predicting pulmonary hypertension in patients with severe COPD. These results suggest an association between impaired lung function and arterial diameter, especially with respect to the pulmonary artery rather than the aorta. Consistent with previous studies, in our study, RVSP determined by echocardiography did not show any significant results, while FEV_1_ (%) negatively correlated with mPA. However, further large-scale studies are needed to demonstrate the relationship between lung function and mPA and mPA/Ao ratio.

This study had several limitations. First, participants were from a single center and mostly middle aged; thus, the results may not accurately represent the Korean population. However, the Severance Hospital Health Screening Center is affiliated with the Severance Hospital, a tertiary hospital that admits patients from all over South Korea. Second, the healthy reference group made up less than half of the total participants, and was predominantly female. However, the number of participants included was not less than in previous studies, and ours is the first such study from Korea [6,7,18]. Third, cardiac or pulmonary symptoms that may be related to mPA and Ao were not assessed. Nonetheless, specific cardiopulmonary symptoms were unlikely in our study group, because most were healthy check-up subjects. However, the subjects might have specific reasons such as other comorbidities or risk factors for undertaking chest CT scan. Therefore, we defined the healthy reference group as those who were free of cardiopulmonary diseases and their risk factors, without abnormal pulmonary CT findings. Lastly, because we based our findings on non-contrast CT images, the measured values would show minor differences compared with those determined on contrast CT. However, ECG-gated, coronary-calcium-scoring CT provides consistent results, and thus allows the accurate measurement of arterial diameter.

## Conclusions

In this study, age- and sex-specific reference values were obtained for mPA and Ao in a population of healthy Koreans. These two, readily available measurements were associated with age, sex, BSA, pulmonary function, and comorbidities such as obesity, hypertension, DM, and dyslipidemia. Currently, regular check-ups that include low-dose lung CT are more frequently performed, especially in individuals at high risk for pulmonary disease. Our reference values provide physicians with clinical clues and information on the current cardiopulmonary status of patients in whom these two parameters are determined on chest CT.

## Supporting Information

S1 Table Original data for study subjects. (XLSX)

S1 Fig Distribution of age and BMI. Histograms show age and BMI distributions in all (n = 2,547) (A, C) and healthy participants (n = 813) (B, D), respectively (dark gray: male, gray: female). BMI, body mass index.
(TIF)

S2 Fig Correlations of mPA, Ao, and mPA/Ao ratio with age, BSA, and FEV_1_ (%). Plots show correlations of mPA, Ao, and mPA/Ao ratio with age, BSA, and FEV_1_ (%). Significant correlations were observed between age with mPA (*r* = 0.183, *P* < 0.001), Ao (*r* = 0.527, *P* < 0.001), and mPA/Ao ratio (*r* = -0.334, *P* < 0.001). BSA was correlated with mPA (*r* = 0.300, *P* < 0.001) and Ao (*r* = 0.227, *P* < 0.001). FEV_1_ (%) was correlated with mPA (*r* = -0.065, *P* = 0.001) and mPA/Ao ratio (*r* = -0.080, *P* < 0.001). mPA, main pulmonary artery; Ao, aorta; FEV_1_, forced expiratory volume in 1 second.
(TIF)

S3 Fig Correlations of mPA, Ao, and mPA/Ao ratio with LAVI and RVSP. Plots show correlations of mPA, Ao, and mPA/Ao ratio values with LAVI and RVSP. Significant correlations were observed between LAVI with mPA (*r* = 0.196, *P* = 0.001) and Ao (*r* = 0.253, *P* < 0.001). No correlations were observed between RVSP and the three parameters. mPA, main pulmonary artery; Ao, aorta; LAVI, left atrial volume index; RVSP, right ventricular systolic pressure.
(TIF)
