# Supplementary figures and images for: Comparison of CT-Determined Pulmonary Artery Diameter, Aortic Diameter, and Their Ratio in Healthy and Diverse Clinical Conditions
Source: PLoS One. 2015 May 8;10(5):e0126646. doi: 10.1371/journal.pone.0126646 (PMC4425684; doi:10.1371/journal.pone.0126646)

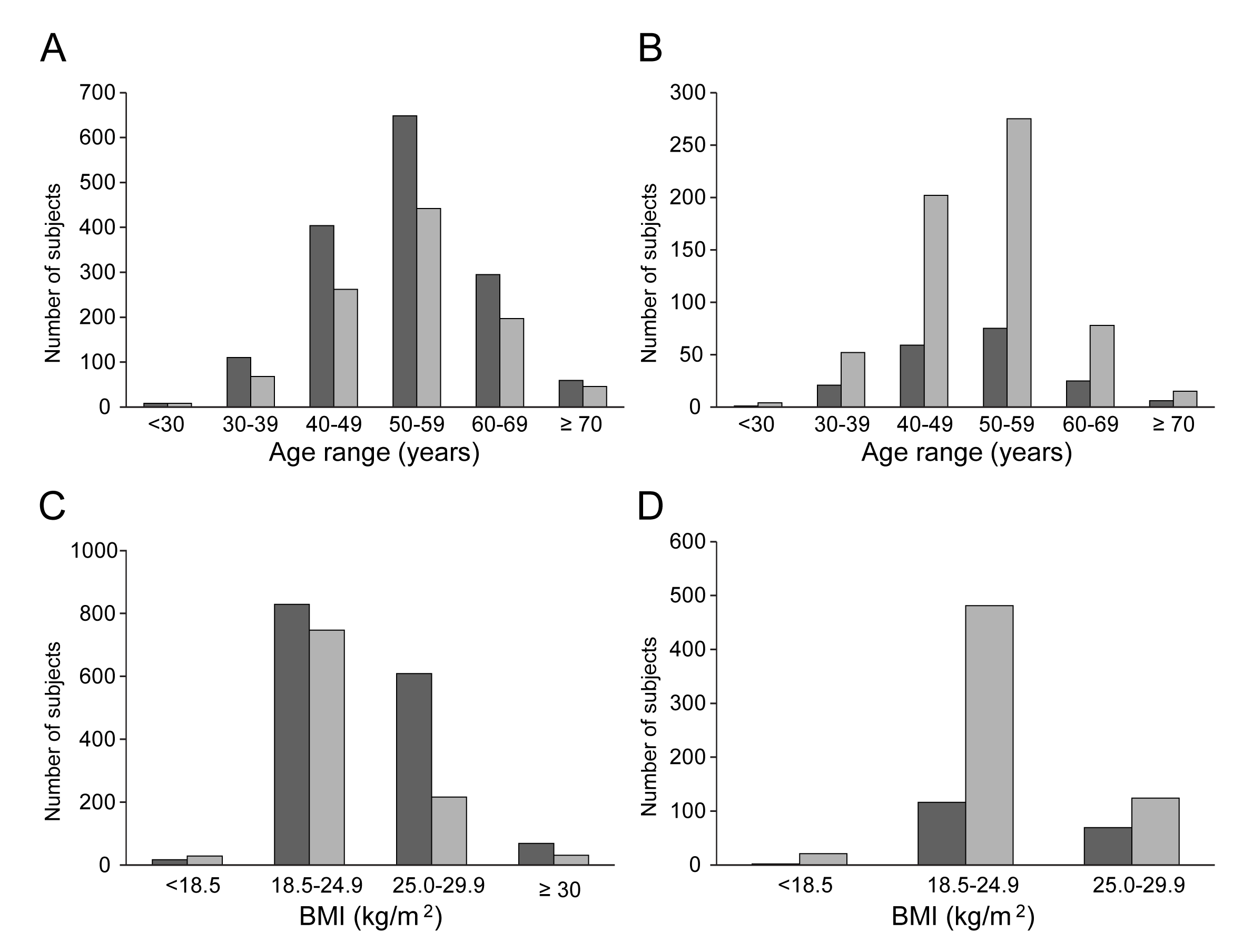

Supplement: S1 Fig — Histograms show age and BMI distributions in all (n = 2,547) (A, C) and healthy participants (n = 813) (B, D), respectively (dark gray: male, gray: female). BMI, body mass index. (TIF) [file pone.0126646.s002.tif]

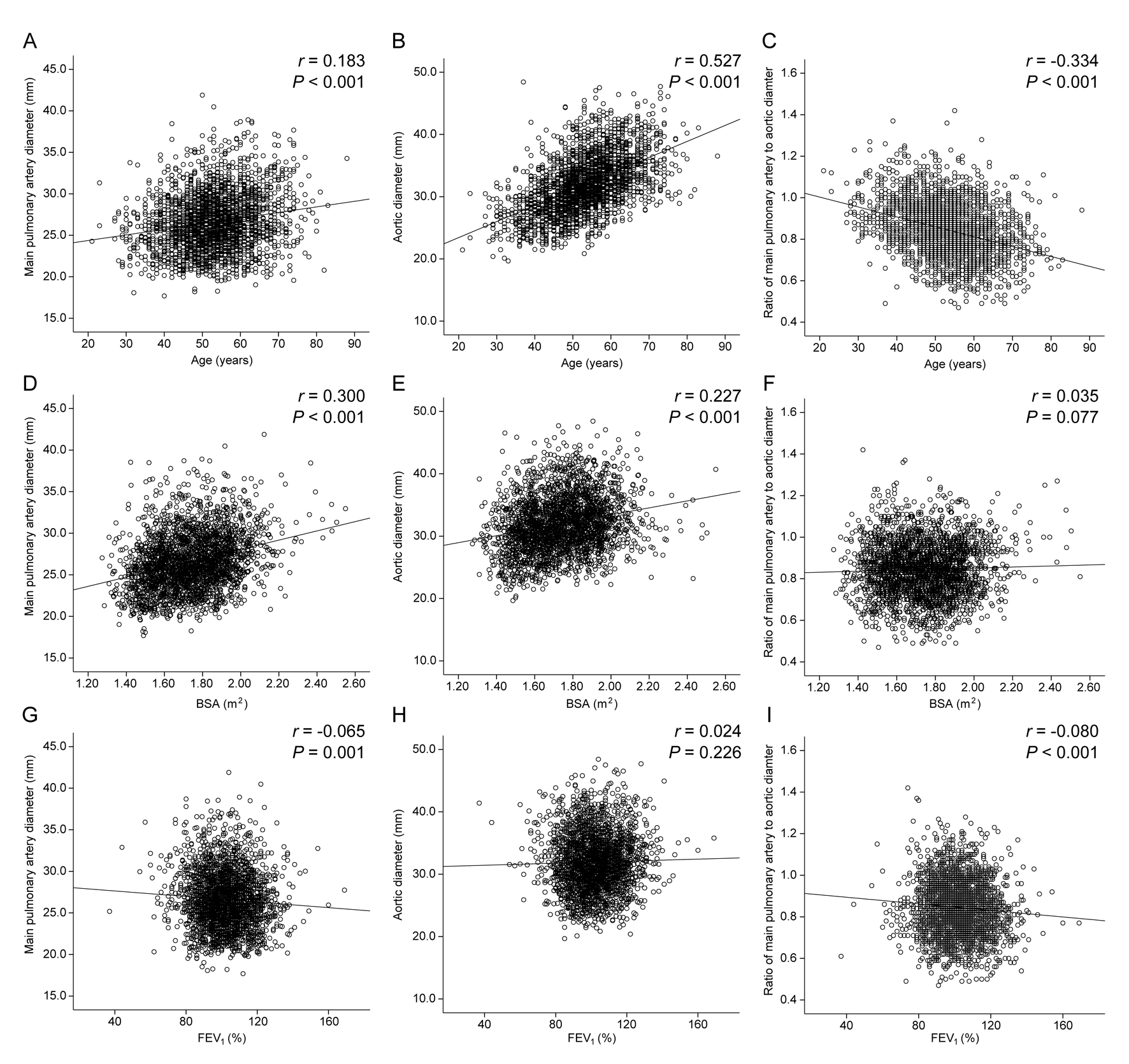

Supplement: S2 Fig — Significant correlations were observed between age with mPA (r = 0.183, P < 0.001), Ao (r = 0.527, P < 0.001), and mPA/Ao ratio (r = -0.334, P < 0.001). BSA was correlated with mPA (r = 0.300, P < 0.001) and Ao (r = 0.227, P < 0.001). FEV1 (%) was correlated with mPA (r = -0.065, P = 0.001) and mPA/Ao ratio (r = -0.080, P < 0.001). mPA, main pulmonary artery; Ao, aorta; FEV1, forced expiratory volume in 1 second. (TIF) [file pone.0126646.s003.tif]

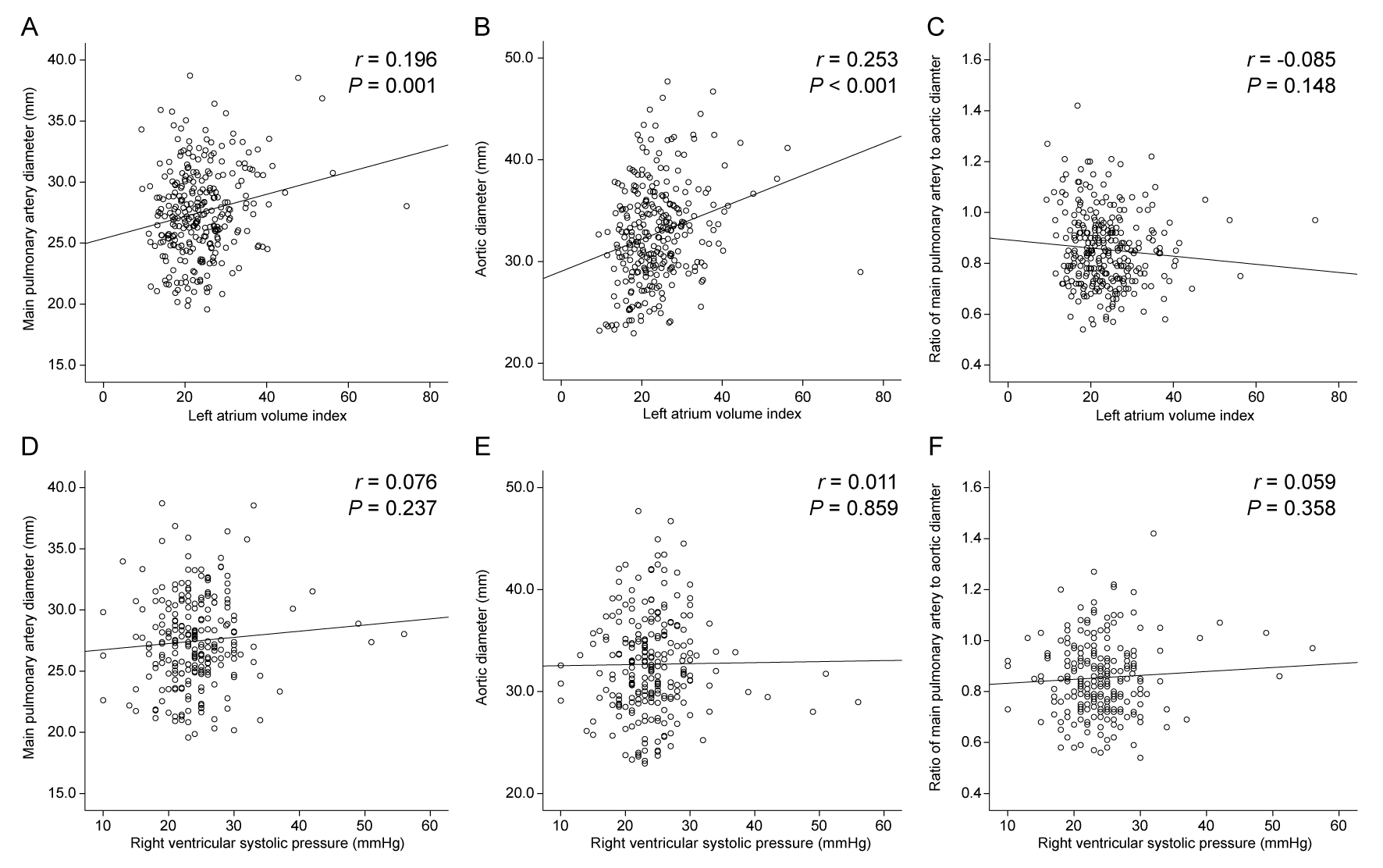

Supplement: S3 Fig — Plots show correlations of mPA, Ao, and mPA/Ao ratio values with LAVI and RVSP. Significant correlations were observed between LAVI with mPA (r = 0.196, P = 0.001) and Ao (r = 0.253, P < 0.001). No correlations were observed between RVSP and the three parameters. mPA, main pulmonary artery; Ao, aorta; LAVI, left atrial volume index; RVSP, right ventricular systolic pressure. (TIF) [file pone.0126646.s004.tif]
